# Supplementary material for: Enhancing the accuracy and efficiency of Pacific walrus (Odobenus rosmarus divergens) surveys: A comparison of visual and aerial imagery-based counts at coastal haulouts
Source: PLoS One. 2024 Jul 16;19(7):e0307416. doi: 10.1371/journal.pone.0307416 (PMC11251640; doi:10.1371/journal.pone.0307416)
Supplement: S1 Table — (DOCX) [file pone.0307416.s001.docx]

## Pairwise comparisons using Wilcoxon rank sum test with continuity correction

##

## data: density and site, ":", count_type

##

## 1001:aerial count 1001:land count 1006:aerial count

## 1001:land count 0.81628 - -

## 1006:aerial count 0.30711 0.83866 -

## 1006:land count 0.00283 0.00025 3e-06

##

## P value adjustment method: holm
