# Supplementary material for: Enhancing the accuracy and efficiency of Pacific walrus (Odobenus rosmarus divergens) surveys: A comparison of visual and aerial imagery-based counts at coastal haulouts
Source: PLoS One. 2024 Jul 16;19(7):e0307416. doi: 10.1371/journal.pone.0307416 (PMC11251640; doi:10.1371/journal.pone.0307416)
Supplement: S2 Table — (DOCX) [file pone.0307416.s002.docx]

## Call:

## MASS::glm.nb(formula = formula, data = density., init.theta = 8.978997196,

## link = log)

##

## Coefficients:

## Estimate Std. Error z value Pr(>|z|)

## (Intercept) -0.43365 0.01119 -38.75 <2e-16 ***

## terrain type 0.30456 0.01949 15.63 <2e-16 ***

## ---

## Signif. codes: 0 '***' 0.001 '**' 0.01 '*' 0.05 '.' 0.1 ' ' 1

##

## (Dispersion parameter for Negative Binomial(8.979) family taken to be 1)

##

## Null deviance: 1871.1 on 1551 degrees of freedom

## Residual deviance: 1616.9 on 1550 degrees of freedom

## AIC: 15395

##

## Number of Fisher Scoring iterations: 1

##

##

## Theta: 8.979

## Std. Err.: 0.379

##

## 2 x log-likelihood: -15389.397
