# Supplementary material for: Enhancing the accuracy and efficiency of Pacific walrus (Odobenus rosmarus divergens) surveys: A comparison of visual and aerial imagery-based counts at coastal haulouts
Source: PLoS One. 2024 Jul 16;19(7):e0307416. doi: 10.1371/journal.pone.0307416 (PMC11251640; doi:10.1371/journal.pone.0307416)
Supplement: S3 Table — Negative values of ratio represent cases when extrapolated value is higher than complete manual count. (DOCX) [file pone.0307416.s003.docx]

| site | date | 2017 | | | 2018 | | | 2019 | | |
| --- | --- | --- | --- | --- | --- | --- | --- | --- | --- | --- |
|  |  | count | extrap | diff | count | extrap | diff | count | extrap | diff |
| KOLYUCHIN | 0831 | 249 | -- | -- | -- | -- | -- | -- | -- | -- |
| KOLYUCHIN | 0901 | 105 | -- | -- | -- | -- | -- | -- | -- | -- |
| KOLYUCHIN | 0903 | 664 | -- | -- | -- | -- | -- | -- | -- | -- |
| KOLYUCHIN | 0906 | 1097 | -- | -- | -- | -- | -- | -- | -- | -- |
| KOLYUCHIN | 0907 | 1417 | -- | -- | -- | -- | -- | -- | -- | -- |
| KOLYUCHIN | 0918 | 164 | -- | -- | -- | -- | -- | -- | -- | -- |
| KOLYUCHIN | 0919 | 1230 | -- | -- | -- | -- | -- | -- | -- | -- |
| KOLYUCHIN | 0920 | 1724 | -- | -- | -- | -- | -- | -- | -- | -- |
| KOLYUCHIN | 0921 | 2976 | -- | -- | -- | -- | -- | -- | -- | -- |
| KOLYUCHIN | 0922 | 2497 | -- | -- | -- | -- | -- | -- | -- | -- |
| KOLYUCHIN | 0923 | 415 | -- | -- | -- | -- | -- | -- | -- | -- |
| KOLYUCHIN | 0926 | 15 | -- | -- | -- | -- | -- | -- | -- | -- |
| KOLYUCHIN | 0927 | 391 | -- | -- | -- | -- | -- | -- | -- | -- |
| KOLYUCHIN | 0928 | 19 | -- | -- | -- | -- | -- | -- | -- | -- |
| LORINO | 1117 | 773 | -- | -- | -- | -- | -- | -- | -- | -- |
| KENISKIN BAY (MAIN) | 0926 | 5216 | -- | -- | -- | -- | -- | -- | -- | -- |
| KENISKIN BAY (MAIN) | 0927 | 14776 | -- | -- | -- | -- | -- | -- | -- | -- |
| KENISKIN BAY (MAIN) | 0929 | -- | -- | -- | -- | -- | -- | -- | 20103 | -- |
| KENISKIN BAY (MAIN) | 1002 | -- | -- | -- | -- | -- | -- | -- | 29641 | -- |
| KENISKIN BAY (MAIN) | 1003 | 24486 | -- | -- | -- | -- | -- | -- | 22845 | -- |
| KENISKIN BAY (MAIN) | 1004 | 26207 | -- | -- | -- | -- | -- | -- | -- | -- |
| KENISKIN BAY (MAIN) | 1005 | 48958 | -- | -- | -- | -- | -- | -- | -- | -- |
| KENISKIN BAY (MAIN) | 1007 | 15836 | -- | -- | -- | -- | -- | -- | 18695 | -- |
| KENISKIN BAY (MAIN) | 1009 | 25609 | -- | -- | -- | -- | -- | -- | -- | -- |
| KENISKIN BAY (MAIN) | 1010 | 13866 | -- | -- | -- | -- | -- | -- | -- | -- |
| KENISKIN BAY (MAIN) | 1011 | -- | -- | -- | -- | -- | -- | -- | 41609 | -- |
| KENISKIN BAY (MAIN) | 1013 | -- | -- | -- | -- | -- | -- | -- | 35849 | -- |
| KENISKIN BAY (MAIN) | 1014 | 7220 | -- | -- | -- | -- | -- | -- | -- | -- |
| KENISKIN BAY (MAIN) | 1015 | 11660 | -- | -- | -- | 26851 | -- | -- | -- | -- |
| KENISKIN BAY (MAIN) | 1016 | 15755 | -- | -- | -- | -- | -- | -- | -- | -- |
| KENISKIN BAY (MAIN) | 1017 | 48830 | -- | -- | -- | -- | -- | -- | -- | -- |
| KENISKIN BAY (MAIN) | 1018 | 94961 | 92474 | 2.62% | -- | -- | -- | -- | -- | -- |
| KENISKIN BAY (MAIN) | 1019 | 65768 | -- | -- | -- | -- | -- | -- | 26826 | -- |
| KENISKIN BAY (MAIN) | 1021 | -- | -- | -- | -- | -- | -- | -- | 57440 | -- |
| KENISKIN BAY (MAIN) | 1022 | -- | -- | -- | -- | -- | -- | -- | 71240 | -- |
| KENISKIN BAY (MAIN) | 1023 | -- | -- | -- | -- | -- | -- | -- | 87596 | -- |
| KENISKIN BAY (MAIN) | 1024 | -- | -- | -- | -- | 10594 | -- | -- | -- | -- |
| KENISKIN BAY (MAIN) | 1031 | -- | -- | -- | -- | 17881 | -- | -- | -- | -- |
| KENISKIN BAY (MAIN) | 1103 | -- | -- | -- | -- | 3454 | -- | -- | -- | -- |
| KENISKIN BAY (MAIN) | 1118 | -- | -- | -- | -- | 7647 | -- | -- | -- | -- |
| KENISKIN BAY (SOUTH) | 1013 | -- | -- | -- | -- | -- | -- | -- | 14361 | -- |
| KENISKIN BAY (SOUTH) | 1021 | -- | -- | -- | -- | -- | -- | -- | 9308 | -- |
| KENISKIN BAY (SOUTH) | 1022 | -- | -- | -- | -- | -- | -- | -- | 14326 | -- |
| KENISKIN BAY (SOUTH) | 1023 | -- | -- | -- | -- | -- | -- | -- | 10270 | -- |
| SERDCE KAMEN CAPE | 0911 | 1239 | 1077 | 13.09% | -- | -- | -- | -- | -- | -- |
| SERDCE KAMEN CAPE | 0914 | 4528 | 4120 | 9.03% | -- | -- | -- | -- | -- | -- |
| SERDCE KAMEN CAPE | 0916 | 6659 | -- | -- | -- | -- | -- | -- | -- | -- |
| SERDCE KAMEN CAPE | 0918 | 5175 | -- | -- | -- | -- | -- | -- | -- | -- |
| SERDCE KAMEN CAPE | 0919 | 11084 | 8611 | 22.31% | -- | -- | -- | -- | -- | -- |
| SERDCE KAMEN CAPE | 0921 | 11067 | -- | -- | -- | -- | -- | -- | -- | -- |
| SERDCE KAMEN CAPE | 0922 | 5386 | -- | -- | -- | -- | -- | -- | 5822 | -- |
| SERDCE KAMEN CAPE | 0926 | 3661 | -- | -- | -- | -- | -- | -- | 6644 | -- |
| SERDCE KAMEN CAPE | 0927 | 5464 | -- | -- | -- | -- | -- | -- | -- | -- |
| SERDCE KAMEN CAPE | 1004 | 4180 | -- | -- | -- | -- | -- | -- | -- | -- |
| SERDCE KAMEN CAPE | 1009 | 2057 | -- | -- | -- | -- | -- | -- | -- | -- |
| SERDCE KAMEN CAPE | 1101 | -- | -- | -- | -- | -- | -- | -- | 2010 | -- |
| SCHMIDT CAPE | 0820 | 499 | -- | -- | -- | -- | -- | -- | -- | -- |
| SCHMIDT CAPE | 0823 | 15 | -- | -- | -- | -- | -- | -- | -- | -- |
| SCHMIDT CAPE | 0826 | 1165 | -- | -- | -- | -- | -- | -- | -- | -- |
| SCHMIDT CAPE | 0829 | 488 | -- | -- | -- | -- | -- | -- | -- | -- |
| SCHMIDT CAPE | 0830 | 1555 | -- | -- | -- | -- | -- | -- | -- | -- |
| SCHMIDT CAPE | 0831 | 1145 | -- | -- | -- | -- | -- | -- | -- | -- |
| SCHMIDT CAPE | 0903 | 352 | -- | -- | -- | -- | -- | -- | -- | -- |
| SCHMIDT CAPE | 0905 | 2007 | -- | -- | -- | -- | -- | -- | -- | -- |
| SCHMIDT CAPE | 0906 | 2416 | -- | -- | -- | -- | -- | -- | -- | -- |
| SCHMIDT CAPE | 0907 | 4676 | -- | -- | -- | -- | -- | -- | -- | -- |
| SCHMIDT CAPE | 0908 | 6409 | -- | -- | -- | -- | -- | -- | -- | -- |
| SCHMIDT CAPE | 0909 | 110 | -- | -- | -- | -- | -- | -- | -- | -- |
| SCHMIDT CAPE | 0910 | 51 | -- | -- | -- | -- | -- | -- | -- | -- |
| SCHMIDT CAPE | 0911 | 819 | -- | -- | -- | -- | -- | -- | -- | -- |
| SCHMIDT CAPE | 0912 | 4150 | -- | -- | -- | -- | -- | -- | -- | -- |
| SCHMIDT CAPE | 0913 | 8103 | -- | -- | -- | -- | -- | -- | -- | -- |
| SCHMIDT CAPE | 0914 | 1818 | -- | -- | -- | -- | -- | -- | -- | -- |
| SCHMIDT CAPE | 0915 | 1815 | -- | -- | -- | -- | -- | -- | -- | -- |
| SCHMIDT CAPE | 0916 | 4307 | -- | -- | -- | -- | -- | -- | -- | -- |
| SCHMIDT CAPE | 0917 | 4272 | -- | -- | -- | -- | -- | -- | -- | -- |
| SCHMIDT CAPE | 0918 | 998 | -- | -- | -- | -- | -- | -- | -- | -- |
| SCHMIDT CAPE | 0919 | 1963 | -- | -- | -- | -- | -- | -- | -- | -- |
| SCHMIDT CAPE | 0920 | 1290 | -- | -- | -- | -- | -- | -- | -- | -- |
| SCHMIDT CAPE | 0921 | 1450 | -- | -- | -- | -- | -- | -- | -- | -- |
| SCHMIDT CAPE | 0922 | 2077 | -- | -- | -- | -- | -- | -- | -- | -- |
| SCHMIDT CAPE | 0923 | 740 | -- | -- | -- | -- | -- | -- | -- | -- |
| SCHMIDT CAPE | 0924 | 226 | -- | -- | -- | -- | -- | -- | -- | -- |
| SCHMIDT CAPE | 0925 | 327 | -- | -- | -- | -- | -- | -- | -- | -- |
| SCHMIDT CAPE | 0927 | 2138 | -- | -- | -- | -- | -- | -- | -- | -- |
| SCHMIDT CAPE | 0928 | 5678 | -- | -- | -- | -- | -- | -- | -- | -- |
| SCHMIDT CAPE | 1003 | 37 | -- | -- | -- | -- | -- | -- | -- | -- |
| INCHOUN | 0914 | 83 | -- | -- | -- | -- | -- | -- | -- | -- |
| INCHOUN | 0928 | 1026 | -- | -- | -- | -- | -- | -- | -- | -- |
| INCHOUN | 1005 | 441 | -- | -- | 251 | -- | -- | -- | -- | -- |
| INCHOUN | 1006 | -- | -- | -- | 538 | -- | -- | -- | -- | -- |
| INCHOUN | 1015 | 1260 | -- | -- | -- | -- | -- | -- | -- | -- |
| INCHOUN | 1016 | 3121 | -- | -- | -- | -- | -- | -- | -- | -- |
| INCHOUN | 1018 | 176 | -- | -- | -- | -- | -- | -- | -- | -- |
| INCHOUN | 1020 | -- | -- | -- | 169 | -- | -- | -- | -- | -- |
| INCHOUN | 1021 | -- | -- | -- | 3252 | -- | -- | -- | -- | -- |
| VANKAREM | 0830 | -- | -- | -- | 2556 | 2840 | -11.09% | -- | -- | -- |
| VANKAREM | 0831 | -- | -- | -- | 3122 | 3092 | 0.95% | -- | -- | -- |
| VANKAREM | 0901 | -- | -- | -- | 2622 | 3027 | -15.44% | -- | -- | -- |
| VANKAREM | 0903 | 6695 | -- | -- | 1907 | 1699 | 10.92% | -- | -- | -- |
| VANKAREM | 0905 | -- | -- | -- | 655 | 750 | -14.52% | -- | -- | -- |
| VANKAREM | 0906 | 9127 | -- | -- | 2054 | 2090 | -1.76% | -- | -- | -- |
| VANKAREM | 0907 | 12986 | -- | -- | 3110 | 3185 | -2.43% | -- | -- | -- |
| VANKAREM | 0908 | -- | -- | -- | 3603 | 3919 | -8.78% | -- | -- | -- |
| VANKAREM | 0909 | -- | -- | -- | 2455 | 3007 | -22.52% | -- | -- | -- |
| VANKAREM | 0910 | 5457 | -- | -- | 2629 | 2799 | -6.47% | -- | -- | -- |
| VANKAREM | 0911 | 10086 | -- | -- | 3016 | 2921 | 3.16% | -- | -- | -- |
| VANKAREM | 0912 | 24548 | -- | -- | 2926 | 3118 | -6.57% | -- | -- | -- |
| VANKAREM | 0913 | -- | -- | -- | 3473 | 3512 | -1.13% | -- | -- | -- |
| VANKAREM | 0914 | 23997 | -- | -- | 3942 | 3954 | -0.31% | -- | -- | -- |
| VANKAREM | 0915 | 19175 | -- | -- | 2034 | 1849 | 9.09% | -- | -- | -- |
| VANKAREM | 0916 | 19683 | -- | -- | 821 | 910 | -10.83% | -- | -- | -- |
| VANKAREM | 0917 | -- | -- | -- | 1952 | 2168 | -11.06% | -- | -- | -- |
| VANKAREM | 0918 | -- | -- | -- | 2923 | 3164 | -8.25% | -- | -- | -- |
| VANKAREM | 0919 | -- | -- | -- | 2928 | 3012 | -2.88% | -- | -- | -- |
| VANKAREM | 0920 | -- | -- | -- | 3196 | 3315 | -3.71% | -- | -- | -- |
| VANKAREM | 0921 | -- | -- | -- | 3549 | 3742 | -5.45% | -- | -- | -- |
| VANKAREM | 0922 | 19748 | -- | -- | 3710 | 3704 | 0.15% | -- | -- | -- |
| VANKAREM | 0923 | 18719 | -- | -- | 2721 | 2915 | -7.12% | -- | -- | -- |
| VANKAREM | 0924 | 15093 | -- | -- | 4514 | 4739 | -4.99% | -- | -- | -- |
| VANKAREM | 0925 | 17234 | 17053 | 1.05% | 7460 | 7386 | 0.99% | -- | -- | -- |
| VANKAREM | 0926 | 11862 | -- | -- | 11838 | 11738 | 0.85% | -- | -- | -- |
| VANKAREM | 0927 | 11762 | 9137 | 22.32% | 20088 | 20572 | -2.41% | -- | -- | -- |
| VANKAREM | 0928 | 26156 | -- | -- | 22532 | 21725 | 3.58% | -- | -- | -- |
| VANKAREM | 0929 | 22119 | -- | -- | -- | -- | -- | -- | -- | -- |
| VANKAREM | 0930 | 10464 | -- | -- | 10965 | 10621 | 3.13% | -- | -- | -- |
| VANKAREM | 1001 | 13176 | 11128 | 15.55% | 16494 | 16494 | 0.00% | -- | -- | -- |
| VANKAREM | 1002 | -- | -- | -- | -- | 18821 | -- | -- | -- | -- |
| VANKAREM | 1003 | 10291 | -- | -- | -- | 13154 | -- | -- | -- | -- |
| VANKAREM | 1004 | 7966 | 7446 | 6.53% | -- | 12111 | -- | -- | -- | -- |
| VANKAREM | 1005 | 6522 | 6276 | 3.78% | -- | 12860 | -- | -- | -- | -- |
| VANKAREM | 1006 | -- | -- | -- | -- | 11520 | -- | -- | -- | -- |
| VANKAREM | 1007 | -- | -- | -- | -- | 5169 | -- | -- | -- | -- |
| VANKAREM | 1008 | 3784 | -- | -- | -- | 16599 | -- | -- | -- | -- |
| VANKAREM | 1009 | 6269 | 6254 | 0.23% | -- | 23798 | -- | -- | -- | -- |
| VANKAREM | 1010 | -- | -- | -- | -- | 15731 | -- | -- | -- | -- |
| VANKAREM | 1011 | 2997 | 3002 | -0.16% | 10380 | 10046 | 3.22% | -- | -- | -- |
| VANKAREM | 1012 | 2236 | -- | -- | 6325 | 5639 | 10.85% | -- | -- | -- |
| VANKAREM | 1013 | 100 | -- | -- | -- | 14227 | -- | -- | -- | -- |
| VANKAREM | 1014 | -- | -- | -- | -- | 16537 | -- | -- | -- | -- |
| VANKAREM | 1015 | -- | -- | -- | -- | 17158 | -- | -- | -- | -- |
| VANKAREM | 1018 | -- | -- | -- | 1388 | 1399 | -0.78% | -- | -- | -- |
| VANKAREM | 1019 | -- | -- | -- | -- | 4615 | -- | -- | -- | -- |
| VANKAREM | 1020 | -- | -- | -- | -- | 7926 | -- | -- | -- | -- |
| VANKAREM | 1022 | -- | -- | -- | -- | 7842 | -- | -- | -- | -- |
| VANKAREM | 1023 | -- | -- | -- | -- | 6744 | -- | -- | -- | -- |
| VANKAREM | 1024 | -- | -- | -- | -- | 3682 | -- | -- | -- | -- |
