# Supplementary figures and images for: Enhancing the accuracy and efficiency of Pacific walrus (Odobenus rosmarus divergens) surveys: A comparison of visual and aerial imagery-based counts at coastal haulouts
Source: PLoS One. 2024 Jul 16;19(7):e0307416. doi: 10.1371/journal.pone.0307416 (PMC11251640; doi:10.1371/journal.pone.0307416)

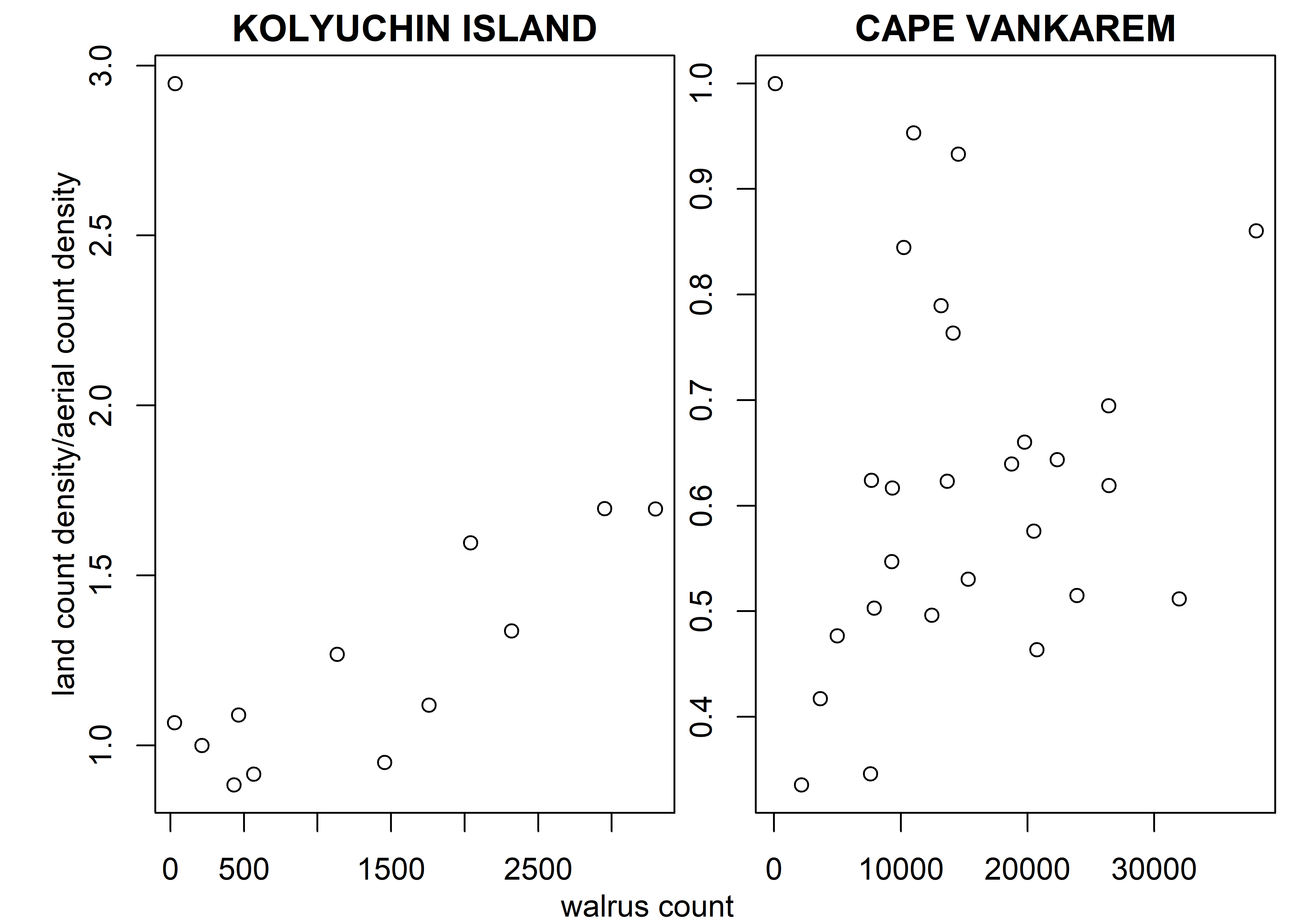

Supplement: S1 Fig — (TIF) [file pone.0307416.s004.tif]

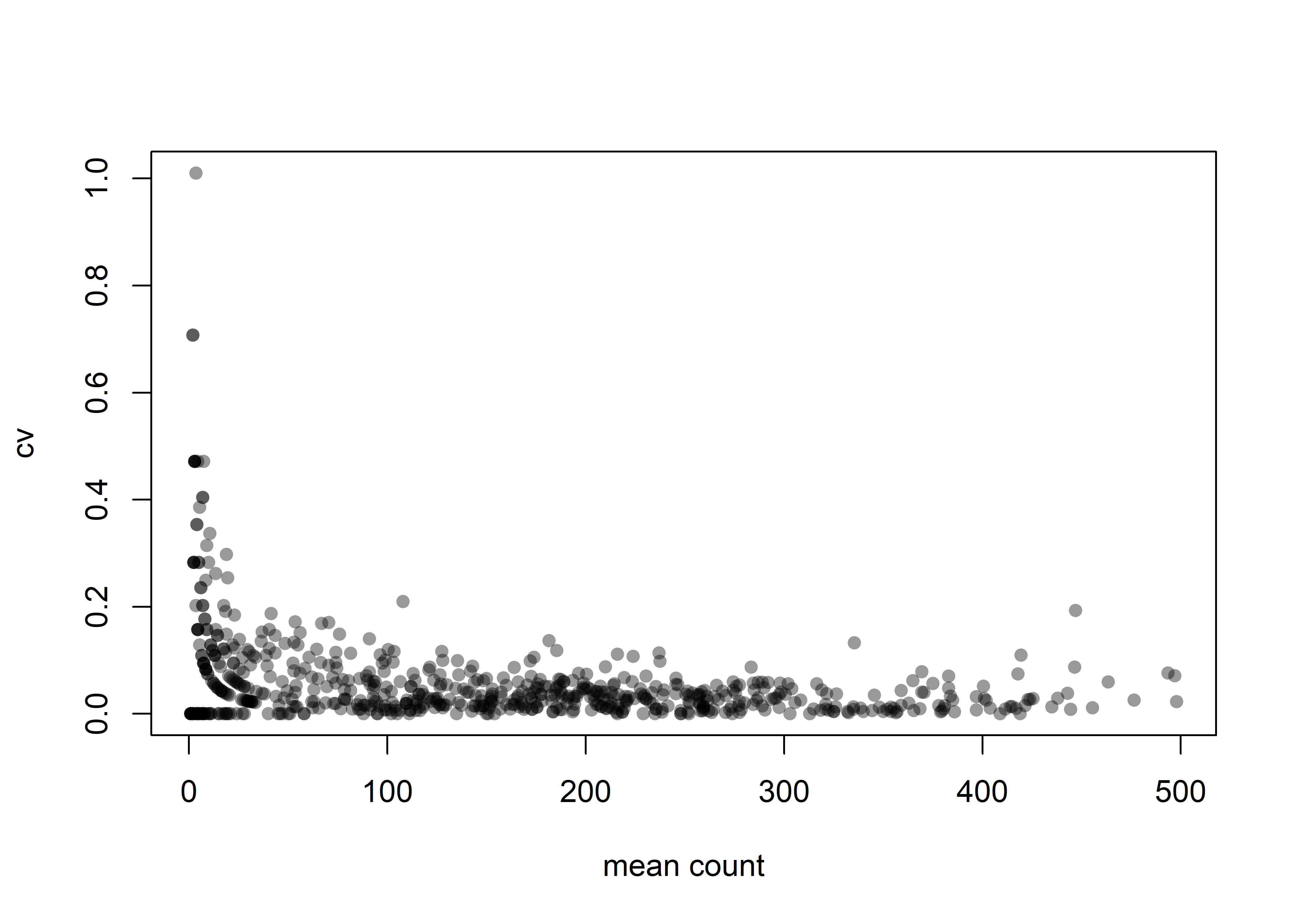

Supplement: S2 Fig — (TIF) [file pone.0307416.s005.tif]

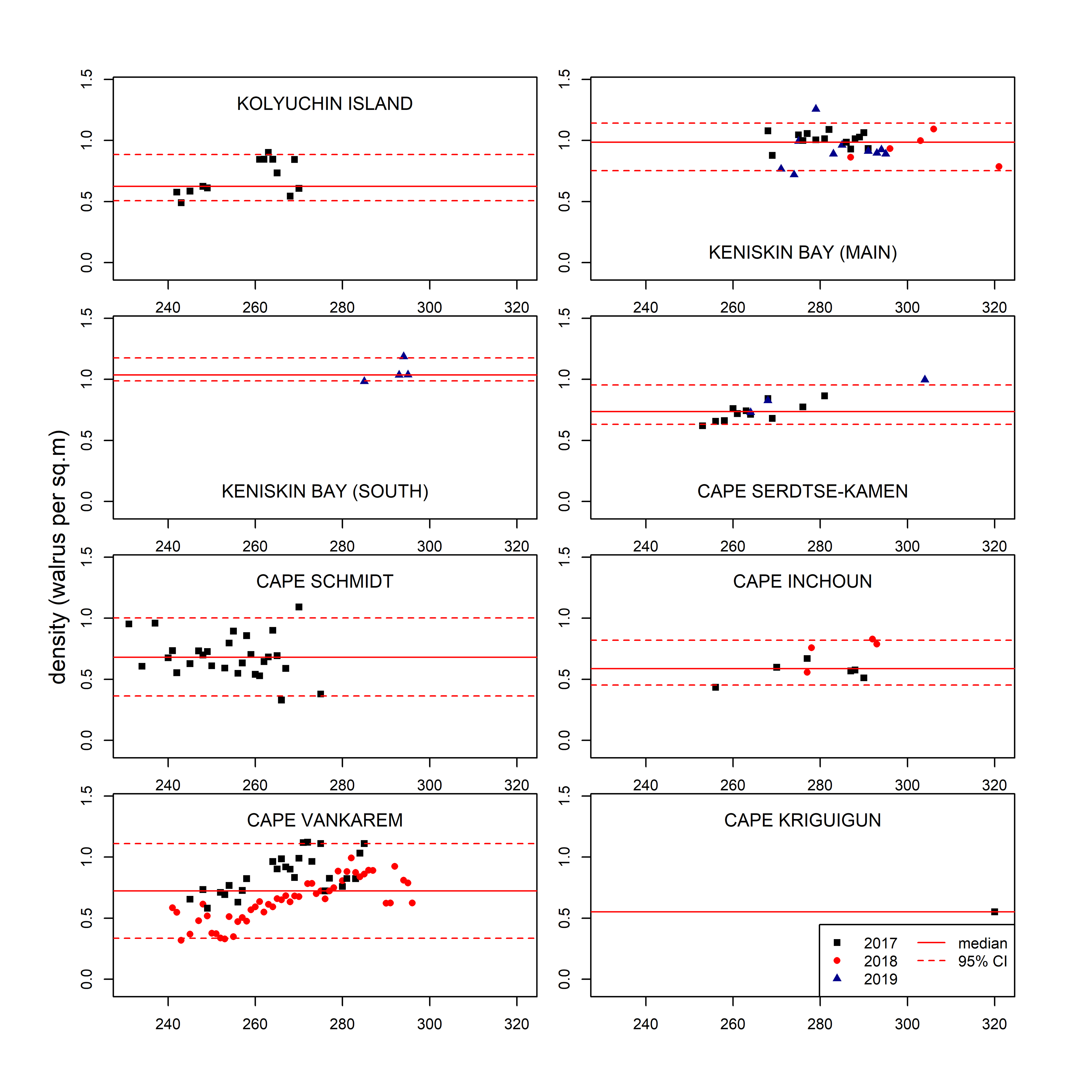

Supplement: S3 Fig — x-axis is a day of the year, y-axis is density in walrus per m2. (TIF) [file pone.0307416.s006.tif]

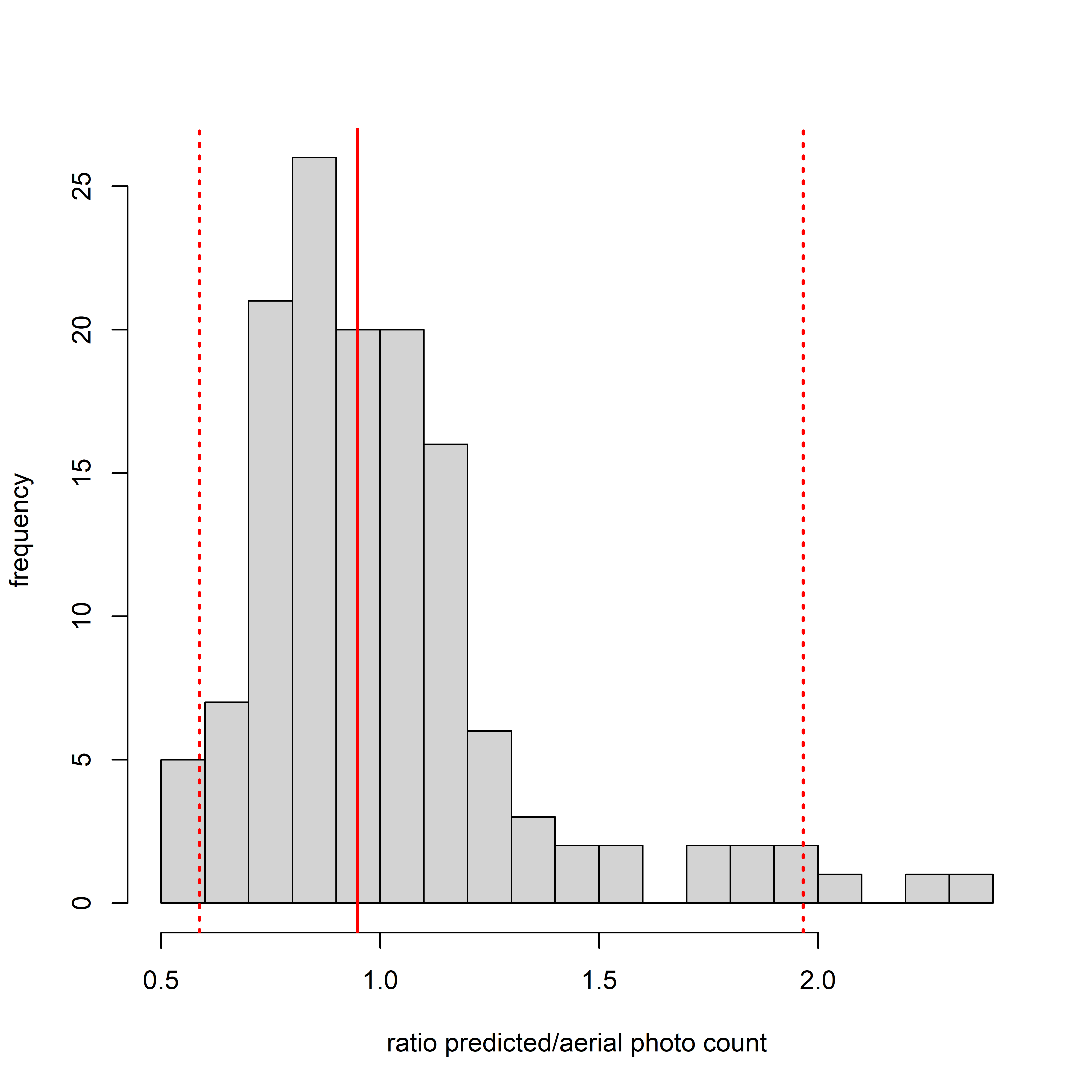

Supplement: S4 Fig — (TIF) [file pone.0307416.s007.tif]

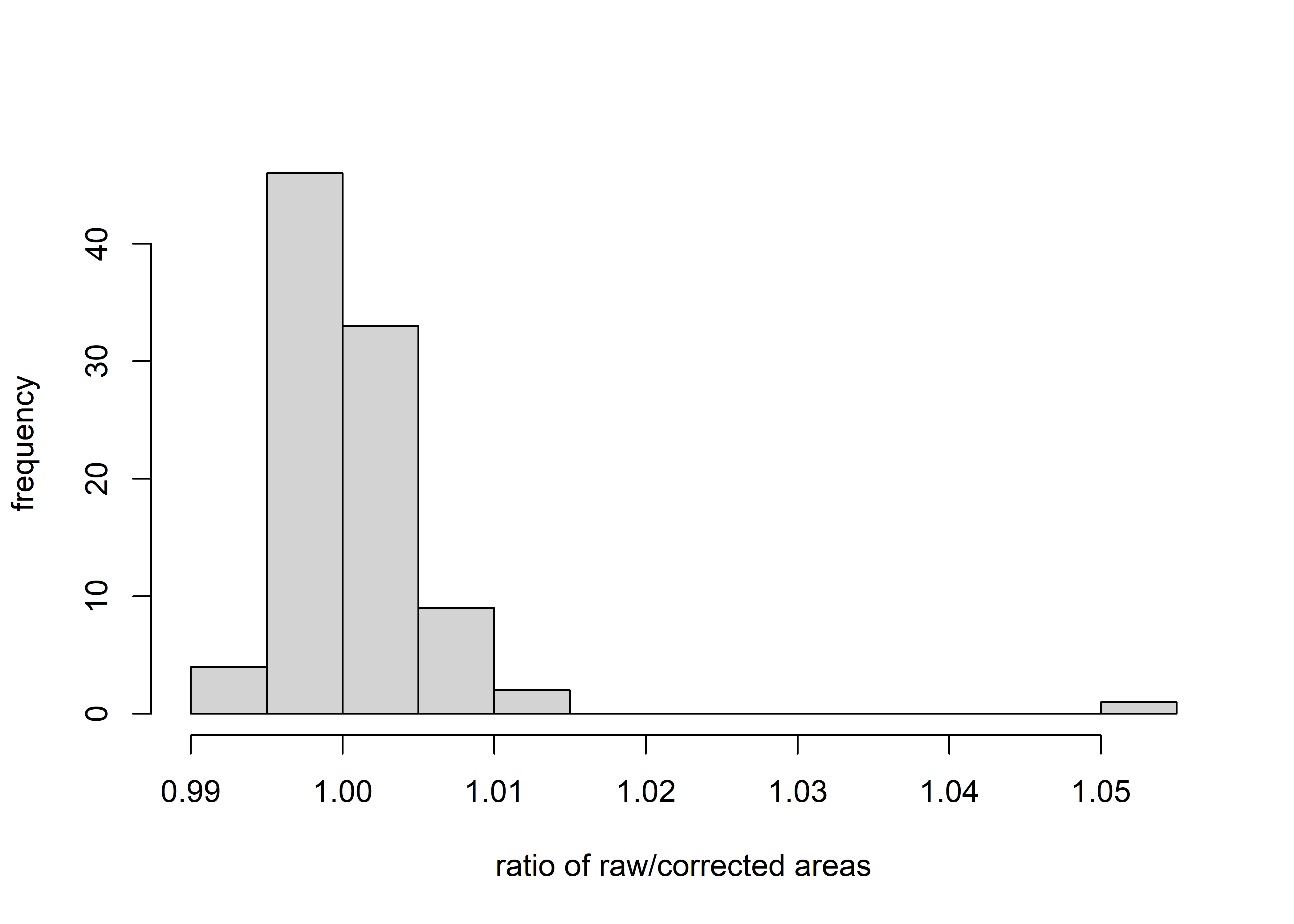

Supplement: S5 Fig — (TIF) [file pone.0307416.s008.tif]
