# Supplementary material for: Enhancing the accuracy and efficiency of Pacific walrus (Odobenus rosmarus divergens) surveys: A comparison of visual and aerial imagery-based counts at coastal haulouts
Source: PLoS One. 2024 Jul 16;19(7):e0307416. doi: 10.1371/journal.pone.0307416 (PMC11251640; doi:10.1371/journal.pone.0307416)
Supplement: S1 Text — (DOCX) [file pone.0307416.s009.docx]

In this study, we did not integrate sparse point cloud gradual selection algorithms recommended in USGS publications into all the mosaics used. Nevertheless, we conducted a test on a specific set of mosaics, applying the gradual selection algorithm outlined in Fischbach et al. (2021) and Logan et al. (2022). Subsequently, we manually created polygons using the same visible features present in both mosaics, one with gradual selection applied and another without. We assessed a total of 12 pairs of mosaics, comparing the areas of 95 pairs of polygons as a ratio of raw areas to those on mosaics with gradual selection applied (refer to S6 Figure). The mean difference in areas was minimal, at less than one percent (-0.11%), indicating slightly larger areas on mosaics without gradual selection. The 95% confidence intervals ranged from 0.57% to - 1.02%.
