# Supplementary material for: Enhancing the accuracy and efficiency of Pacific walrus (Odobenus rosmarus divergens) surveys: A comparison of visual and aerial imagery-based counts at coastal haulouts
Source: PLoS One. 2024 Jul 16;19(7):e0307416. doi: 10.1371/journal.pone.0307416 (PMC11251640; doi:10.1371/journal.pone.0307416)
Supplement: Attachment — Submitted filename: Response to Reviewers.docx [file pone.0307416.s011.docx]

PONE-D-24-08990

Enhancing the accuracy and efficiency of Pacific walrus (Odobenus rosmarus divergens) surveys: a comparison of visual and aerial imagery-based counts at coastal haulouts.

PLOS ONE

Dear Dr. Altukhov,

Thank you for submitting your manuscript to PLOS ONE, and thank you for your patience while I identified two reviewers who were able to provide reviews of the manuscript. Both reviewers agreed that the manuscript provided significant contributions to enumerating walruses in coastal haul-outs. Nevertheless both reviewers provided editing suggestions and posed a few questions that would be helpful to clarify in a revised version of the manuscript. Therefore, after careful consideration, we invite you to submit a revised version of the manuscript that addresses the points raised during the review process.

Please submit your revised manuscript by Jun 20 2024 11:59PM. If you will need more time than this to complete your revisions, please reply to this message or contact the journal office at plosone@plos.org. When you're ready to submit your revision, log on to https://www.editorialmanager.com/pone/ and select the 'Submissions Needing Revision' folder to locate your manuscript file.

Please include the following items when submitting your revised manuscript:

A rebuttal letter that responds to each point raised by the academic editor and reviewer(s). You should upload this letter as a separate file labeled 'Response to Reviewers'.

A marked-up copy of your manuscript that highlights changes made to the original version. You should upload this as a separate file labeled 'Revised Manuscript with Track Changes'.

An unmarked version of your revised paper without tracked changes. You should upload this as a separate file labeled 'Manuscript'.

If you would like to make changes to your financial disclosure, please include your updated statement in your cover letter. Guidelines for resubmitting your figure files are available below the reviewer comments at the end of this letter.

If applicable, we recommend that you deposit your laboratory protocols in protocols.io to enhance the reproducibility of your results. Protocols.io assigns your protocol its own identifier (DOI) so that it can be cited independently in the future. For instructions see: https://journals.plos.org/plosone/s/submission-guidelines#loc-laboratory-protocols. Additionally, PLOS ONE offers an option for publishing peer-reviewed Lab Protocol articles, which describe protocols hosted on protocols.io. Read more information on sharing protocols at https://plos.org/protocols?utm_medium=editorial-email&utm_source=authorletters&utm_campaign=protocols.

We look forward to receiving your revised manuscript.

Kind regards,

Lee W Cooper, Ph.D.

Section Editor

PLOS ONE

To Lee W Cooper, PhD

Section Editon

PLOW ONE

Dear Dr. Cooper,

We would like to express our sincere gratitude to you, Dr. Evangeline Corcoran, and the anonymous reviewer for thoughtful reviews of our manuscript titled " Enhancing the accuracy and efficiency of Pacific walrus (Odobenus rosmarus divergens) surveys: a comparison of visual and aerial imagery-based counts at coastal haulouts." Your constructive feedback, editorial suggestions, and clarifying questions have greatly contributed to the refinement and improvement of our work.

We have carefully considered each of the reviewers’ comments and have made the necessary revisions to ensure that our manuscript meets the high standards of the journal. This input has not only strengthened the scientific rigor of our work but has also helped to clarify key points for the readership.

We are submitting a revised version of the manuscript along with the Supplemental materials. Below, you will find our comment-by-comment responses to the reviewers in red.

Once again, we thank you for your valuable contributions to our manuscript and we look forward to the possibility of seeing our revised manuscript published in PLOS ONCE.

Sincerely,

Alexey Altukhov

Natalia Kryukova

Irina Trukhanova

Vladimir Burkanov

Journal requirements:

When submitting your revision, we need you to address these additional requirements.

1. Please ensure that your manuscript meets PLOS ONE's style requirements, including those for file naming. The PLOS ONE style templates can be found at

https://journals.plos.org/plosone/s/file?id=wjVg/PLOSOne_formatting_sample_main_body.pdf and

https://journals.plos.org/plosone/s/file?id=ba62/PLOSOne_formatting_sample_title_authors_affiliations.pdf

2. When completing the data availability statement of the submission form, you indicated that you will make your data available on acceptance. We strongly recommend all authors decide on a data sharing plan before acceptance, as the process can be lengthy and hold up publication timelines. Please note that, though access restrictions are acceptable now, your entire data will need to be made freely accessible if your manuscript is accepted for publication. This policy applies to all data except where public deposition would breach compliance with the protocol approved by your research ethics board. If you are unable to adhere to our open data policy, please kindly revise your statement to explain your reasoning and we will seek the editor's input on an exemption. Please be assured that, once you have provided your new statement, the assessment of your exemption will not hold up the peer review process.

*The data if fully available online, and the link to the depository hosting was provided during initial submission.*

3. We note that Figure 1 in your submission contain [map/satellite] images which may be copyrighted. All PLOS content is published under the Creative Commons Attribution License (CC BY 4.0), which means that the manuscript, images, and Supporting Information files will be freely available online, and any third party is permitted to access, download, copy, distribute, and use these materials in any way, even commercially, with proper attribution. For these reasons, we cannot publish previously copyrighted maps or satellite images created using proprietary data, such as Google software (Google Maps, Street View, and Earth). For more information, see our copyright guidelines: http://journals.plos.org/plosone/s/licenses-and-copyright.

*The map on Figure 1 was generated R using several open source packages. The information is updated in captions.*

Reviewer #1: The authors present a comparison of traditional visual counts, manual counts derived from UAV imagery, and semi-automated methods of estimating walrus abundance that is methodologically sound and likely to be of high interest to both ecologists and other conservation decision-makers involved in monitoring and managing walrus species. The manuscript is well-presented overall with some minor errors in grammar and phrasing that should be edited to improve the clarity of the text. Please see specific comments in the attached document for details on suggested changes.

Other than the aforementioned minor language issues, I have only one concern with the manuscript that I believe needs addressing before I can recommend it be published: Throughout the manuscript multiple claims that the semi-automated method is more time efficient compared to complete counts from UAV imagery. This is likely true, but it is hard to understand the scale of difference in efficiency as the authors have not provided more specific results on the time taken to conduct each method. Adding this information to the results or discussion section would not only strengthen the authors conclusions that the semi-automated method is beneficial but would also help inform decision-making on when and where it would be more appropriate to the use the semi-automated or manual count from UAV imagery methods.

*Time required to conduct a photo-based complete head count depends on herd size, image quality (images of higher quality are easier to process) and observer’s experience. On average, it is safe to assume that we can count (put a marker on the image) 1 walrus per second. Therefore, to process a haulout with 10,000 walruses on it, an observer will spend approximately 166 minutes. With polygon-based count and subsequent extrapolation, it takes about 1/3 of that time (i.e., 55 minutes). For comparison’s sake, for larger haulouts with ~100,000 animals, we are looking at 27.6 hours vs 9.2 h of work time.*

*We thank the reviewer for the question and we have added the following language to the discussion:*

*“As an example, counting all the animals at a 10,000 walrus haulout takes approximately 2.7 hours, with one walrus being counted per second. However, a semi-automated approach reduces this processing time to just 55 minutes.”*

**Specific Comments**

Line 42 – Use of ‘behavioural studies’ and ‘behaviour’ in the same sentence seems redundant, I suggest this line be revised to be more succinct.

*Replaced* with biological studies

Line 50 – Change ‘speed of method’ to ‘speed of the method’.

Done

Line 51 – It would be more concise to say the counts can differ by up to 30% compared to the true number of walruses present.

*We feel that the current wording is very clear and reflects the detected deviations in traditional count results from true values of walrus abundance*

Line 70 – Change ‘allows to obtain’ to ‘allows obtainment of’.

*Changed to allows obtaining….*

Table 1 – It is unclear why some values are missing from this table. Please provide an explanation in the text introducing the table or the table caption.

*Added: Empty cells indicate that a particular type of count was not performed for that location and year.*

Line 121 – It would be helpful to include more information on why drones were flow at different heights and using different flight modes for certain surveys.

*The optimum altitude for a drone survey depends on several parameters: 1) how load a drone is (i.e. model) – typically – the louder, the higher we fly; 2) how much background noise there is (the more noise (wind, waves, birds), the lower we can fly without animals noticing the drone; 3) the drone camera capabilities (certain drones have better cameras and therefore allow for higher flights without significant loss of image quality); and 4) terrain (in cliffy locations, the flights were performed at higher altitudes for safety reasons). Considering the large number of simultaneously surveyed sites and limited project budget (as it usually is the case..) various drone models were used for the survey. Flight parameters were adjusted for specific conditions (see 1-4 above) to ensure image quality sufficient for the study needs while taking in to account walrus disturbance tolerance.*

*We provided explanation in the text:*

*Adjustments in flight altitude could be made to ensure optimal image quality across diverse environmental conditions while adhering to walrus tolerance to disturbance and flight safety considerations.*

Table 2 – More information is needed in the table caption to explain which method the various counts and extrapolations were derived from.

*We provided the following clarification: Predictions with lower and upper CI bound are obtained from non-specific negative binomial GLM counts – complete head count data obtained from orthomosaics; extrapolation – model polygon-based extrapolation results.*

Reviewer #2: This is an interesting contribution about how to best enumerate walrus in late summer and fall coastal haulouts. The authors use UAV or satellite imagery as the gold standard and through the time-consuming process of counting individuals specify that as the “actual” number of walruses present. Comparisons with visual estimates by observers show that is likely less accurate, but short cuts taken by devising polygons and extrapolations can provide pretty accurate counts also. I think with some minor attention, this manuscript can be improved and made ready for publication. Some of the English, while not bad, can be improved and I provide some recommendation for specific line numbers. Also, it would be worth addressing the importance of making highly accurate counts of animals in haulouts, which was never really justified. While accurate enumeration can enable understanding the overall population of Pacific walruses, if an individual haulout is inaccurately counted by 25 or 30% (line 360), what are the consequences? This should be articulated better. It goes beyond stating that accurate and efficient counts (Line 347) are “paramount.” Please tell us why it is so important to have highly accurate counts.

*The imprecision of visual counts is not the biggest issue, but the inability to assess how imprecise a particular count was, i.e., estimate the uncertainly around the point estimate, is a big problem for any scientific data. Visual counts not only suffer from poor precision but also lack estimates of u**ncertainty around the estimated number of animals. This means that the uncertainty of the count itself cannot be propagated further when using the local herd abundance to estimate population parameters, such as total population abundance, age and sex composition, reproductive rates, or mortality. Additionally, the visual count results obtained in different years are impossible to reliably compare to one another as they are highly dependent on individual observer’s error which is also not getting estimated (but shown to be rather large). This makes visual counts less valuable for population monitoring for research, conservation, or management needs. Model polygon-based counts (and complete imagery-based counts too, of course), on the contrary, allow for estimating precision around point estimates, are verifiable because allow for repeat counts by different observers, and the abundance estimation results can be compared across studies, years of sites. Overall, the biggest advantages model polygon-based counts provide, except for more precise results, are estimable uncertainty and verifiable results.*

*We thank the reviewer for bringing this question up. We added some language to the Introduction and in the Discussion to articulate this better.*

My line by line recommendations for editing:

Line 25-26. The implication of this statement of course is that one approach for estimation, a traditional visual account, is not the definitive enumeration method. The critical word here is “actual” that indicates that there is a single correct number when there is a probably an error associated with each estimation, but I think the authors are correct that the best estimates are obtained by the time-consuming task of counting individual animals on a digital photographic image. It would be better to state that the enumeration techniques did not yield the same consistent results as the other two methods, not that it was inherently incorrect.

Line 27-28. Again, I would state that the results here are of consistency, not adherence to a “true” number that is correct.

*The following wording is suggested: The results indicated that traditional visual counts neither yielded consistent results nor allowed for uncertainty estimation, unlike the site- and date-specific direct extrapolation method and the non-specific linear regression model. These latter methods consistently provided estimates, on average, within 5% of the "true" abundance determined through complete photo-based head counts. Beside yielding accurate estimates, these semi-automated methods significantly reduced counting time by at least 63%, in contrast to complete head counts. The non-specific model, which allowed the estimation of walrus abundance based on the type of the terrain and the haulout area was less accurate compared with site and date specific estimates, but provided a tool to estimate abundance when no field visits are conducted, e.g., by using high-resolution satellite imagery to measure haulout area.*

Line 29. Why is it called the universal model? It might be the traditionally used model or the standard model, but universal sounds to all-encompassing.

*Replaced with non-specific. This is in contrast with site specific model.*

Line 42. Not sure what is meant by vital rates.

*By vital rates here we assume information on e.g.,* *birth rate, survival of young, or mortality. Clarified in the text.*

Line 59. Using “or” between UAVs and drones implies that they are different vehicles.

*Corrected: with unoccupied aerial vehicles (UAVs or drones)*

Line 70-72. This sentence is awkwardly written, e.g. “allows to”

*Corrected – allows obtaining*

Line 81. This comes back to my comments on the abstract. The hypothesis is that semi-automated counts using a modeling approach based upon beach topography and haulout size would be more reliable than simply enumerating animals. But how do you assess whether the hypothesis can be accepted? It presumes that there is a gold standard to compare an enumeration method against, doesn’t it?

*Correct. The gold standard is the total head count performed by observers using georeferenced orthomosaics. Repeated full counts performed for selected images showed highly consistent estimates.*

Line 89. Instead of “collected” use “undertook”

*Done*

Line 113. Delete “mentioned” ---it isn’t necessary.

*Deleted*

Line 116. Add the manufacturer’s location (Shenzen, China).

*Added*

Line 138. For the reader not familiar with the software used, it would be helpful to fully spell out and define these parameters, e.g. from https://agisoft.freshdesk.com/support/solutions/articles/31000158119-what-does-camera-calibration-results-mean-in-metashape-:

f - Focal length measured in pixels (in pixels).

cx, cy - Principal point coordinates, i.e. coordinates of lens optical axis interception with sensor plane (in pixels).

b1, b2 - Affinity and non-orthogonality (skew) coefficients (in pixels).

k1, k2, k3, k4 - Radial distortion coefficients (dimensionless).

p1, p2 - Tangential distortion coefficients (dimensionless).

*Thank you for that! Added to text.*

Line 155-157. It is not clear if this was software that the authors designed and wrote themselves, or if it was a modification of an existing product. If it was an existing product that the authors used, give the full name and source of the software. On line 157, the weblink provided does not lead to a working website.

*The software was developed by North Pacific Wildlife Consulting specifically for this and similar projects. We clarified that and we fixed the link.*

Line 189. Insert “the” before Wilcoxon

*Done*

Line 190. This short sentence is not clear. It is stating that there were multiple observers? (Observers should be plural in that case).

*Fixed – observers.*

Line 211. Constructed would probably be a better word that built in this sentence.

*Replaced.*

Line 217. CI was used as early as Line 200, where it should be initially defined instead of here.

*Fixed*

Line 218. Add to “we applied the delta method” “as follows.”

*Done*

Line 221. Use superscript here for exponents rather than ^

*Done*

Line 226. Change “using the R software” to using R

*Done*

Line 233. Use among instead of between

*Done*

Line 235. Add “The” to the start of the sentence. For Figure 2, the numbers on the y-axis should be labelled.

*Done and fixed.*

Line 238. Add the article “a” before tendency.

*Added*

Line 229, 240. Elsewhere, this landmark is referred to as Cape Vankarem rather than Vankarem Cape. Cape Vankarem is probably more familiar to English speakers, but in any case, the geographical landmark should be referred to consistently throughout the paper.

Corrected – now using Cape Vankarem throughout, we also standardized other geographical names throughout the text and figures.

Line 241, 242, 243, 258, 259, 293. Use superscript for exponents.

*Done*

Line 274. Add “the” before extrapolation.

*Done*

Line 275. Change this sentence to “A Summary of extrapolation results with comparison to known complete counts is provided in S1 Table.”

*Changed*

Line 282. Add “the” before walrus
*Added*

Line 285. Not sure what more than one level means. More than one parameter?

*Suggested correction: We refrained from incorporating additional terms into the model, such as the year or month of observation, as most sites were surveyed within a single month or year.*

Line 289. The equation used isn’t clear to me, particularly the tilde (~). Normally this mean “about,” but this seems to be part of the regression equation?

*This is a standard model formulation. This formula represents the count variable as a function of the type variable, with the offset term incorporating the logarithm of the sample area. In this context, the tilde symbol (~) serves as an operator that separates the dependent variable from the independent variables and any other terms in the model.*

Line 298. Spell out generalized linear model on first use (GLM)

*Done*

Line 303. Add the article “the” before “true”

*Done*

Line 326. Change “count” to “amount”

*Changed*

Line 327. Delete “and more”

*Deleted*

Line 330-332. This sentence is redundant with a very similar sentence at Line 40-42. Most of this paragraph presents background information that is also in the introduction.

*Removed*.

Line 337. Change “close to perfect estimate” to “highly accurate estimates”

*Changed to ‘highly precise’*

Line 338 Change “by using multiple observers to process” to “by multiple observers processing”

*Done*

Line 340. Change “a person days to complete the task” to “many days to complete”

*Done*

Line 376. Change “allowing to estimate the” to “facilitating the estimation of”

*Done*

Line 410. Change “provide most precise results” to “provides the objectively most precise results”

*Done*

Line 415 Insert “will be” between “therefore” and “useful”

*Done*

Line 417. Change “in” to “for”

*Done*

References section, for the publications that are written in Russian, state “in Russian” rather than just “Russian.” Species names should be italicized.

*According to The International Committee of Medical Journal Editors (ICMJE) formatting guidelines used by PLOS journals, the current format is correct. They do not italicize species names either. No changes were made.*

Line 520-523. The name of the proceedings is provided twice. Probably helpful here to provide a weblink to where the document is available (<https://library.wcs.org/doi/ctl/view/mid/33065/pubid/DMX1388500000.aspx>)

*Corrected and added, thank you!*

Figure 3. The units for density should be provided on the y-axis itself, not just in the figure caption, i.e., walruses per square meter

*Corrected*

Figure 4. Again, as with the other figures, make sure the axes are clearly labelled as to which type of survey corresponds to each axis and use consistent numbering. Here it is inconsistent with scientific notation used on one graph and ordinal numbers on the other two.

I see the same limited axis labelling in the supplemental figures. On supplemental figure 5, difference is misspelled. On supplemental Figure 4, spell out full Histogram of diff. Axis labels needed on Figure 3 (suppl.), spell out cv on supplemental Figure 2, and on supplemental Figure 1, give the units for density (e.g. walrus per square meter).

*Corrected*
