# Supplementary material for: Enhancing the accuracy and efficiency of Pacific walrus (Odobenus rosmarus divergens) surveys: A comparison of visual and aerial imagery-based counts at coastal haulouts
Source: PLoS One. 2024 Jul 16;19(7):e0307416. doi: 10.1371/journal.pone.0307416 (PMC11251640; doi:10.1371/journal.pone.0307416)
Supplement: Attachment — Submitted filename: Altukov et al. 2024 - Reviewer Comments.docx [file pone.0307416.s010.docx]

**Reviewer Comments – Altukov et al. 2024**

**Manuscript Title: Enhancing the accuracy and efficiency of Pacific walrus (Odobenus rosmarus divergens) surveys: a comparison of visual and aerial imagery-based counts at coastal haulouts.**

**General Comments**

The authors present a comparison of traditional visual counts, manual counts derived from UAV imagery, and semi-automated methods of estimating walrus abundance that is methodologically sound and likely to be of high interest to both ecologists and other conservation decision-makers involved in monitoring and managing walrus species. The manuscript is well-presented overall with some minor errors in grammar and phrasing that should be edited to improve the clarity of the text. Please see specific comments below for details on suggested changes.

Other than the aforementioned minor language issues, I have only one concern with the manuscript that I believe needs addressing before I can recommend it be published: Throughout the manuscript multiple claims that the semi-automated method is more time efficient compared to complete counts from UAV imagery. This is likely true, but it is hard to understand the scale of difference in efficiency as the authors have not provided more specific results on the time taken to conduct each method. Adding this information to the results or discussion section would not only strengthen the authors conclusions that the semi-automated method is beneficial but would also help inform decision-making on when and where it would be more appropriate to the use the semi-automated or manual count from UAV imagery methods.

**Specific Comments**

Line 42 – Use of ‘behavioural studies’ and ‘behaviour’ in the same sentence seems redundant, I suggest this line be revised to be more succinct.

Line 50 – Change ‘speed of method’ to ‘speed of the method’.

Line 51 – It would be more concise to say the counts can differ by up to 30% compared to the true number of walruses present.

Line 70 – Change ‘allows to obtain’ to ‘allows obtainment of’.

Table 1 – It is unclear why some values are missing from this table. Please provide an explanation in the text introducing the table or the table caption.

Line 121 – It would be helpful to include more information on why drones were flow at different heights and using different flight modes for certain surveys.

Table 2 – More information is needed in the table caption to explain which method the various counts and extrapolations were derived from.
